# Application of *Z*-Number Based Modeling in Psychological Research

**DOI:** 10.1155/2015/760403

**Published:** 2015-08-03

**Authors:** Rafik Aliev, Konul Memmedova

**Affiliations:** ^1^Department of Computer Aided Design, Azerbaijan State Oil Academy, Azerbaijan; ^2^Department of Psychological Counselling and Guidance, Near East University, 99138 Lefkoşa, Northern Cyprus, Mersin 10, Turkey

## Abstract

Pilates exercises have been shown beneficial impact on physical, physiological, and mental characteristics of human beings. In this paper, *Z*-number based fuzzy approach is applied for modeling the effect of Pilates exercises on motivation, attention, anxiety, and educational achievement. The measuring of psychological parameters is performed using internationally recognized instruments: Academic Motivation Scale (AMS), Test of Attention (*D*2 Test), and Spielberger's Anxiety Test completed by students. The GPA of students was used as the measure of educational achievement. Application of *Z*-information modeling allows us to increase precision and reliability of data processing results in the presence of uncertainty of input data created from completed questionnaires. The basic steps of *Z*-number based modeling with numerical solutions are presented.

## 1. Set of Problem

The investigation of the effect of physical activities on motivation, attention, anxiety, and educational performances of students is one of the important research areas in psychology. Hannaford in [1] noticed that “Thinking and learning are not all in our head. Our movements that not only express knowledge and facilitate greater cognitive function, they actually grow the brain as they increase in complexity. Our entire brain structure is intimately connected to and grown by the movement mechanism within our body.” Recently some research works, demonstrating the achievement of the academic performances through physical activities, have been published in different sources. In [2] is given a review showing the effectiveness of aerobic, resistance, and multimodal exercise interventions on a wide range of outcome measures, including cognition, general physical function, mobility, strength, balance, flexibility, quality of life. It was shown [3] that the students with the highest fitness level performed better on standardized tests and students with the lowest fitness level performed lower in class grades. In [4–6] it was found that the physical activity improves a cognitive function, learning, and academic achievement. Salmon in [7] presented cross-sectional and longitudinal studies analyzing the effect of physical exercises on anxiety, depression, and sensitivity to stress. Increased physical activity therefore reduces premature mortality [8] and the establishment and maintenance of exercises' habits has become target for clinical psychologists [9].

The above research studies consider impact of psychological activities on performances of students using different psychological parameters. Considering and modeling the effect of physical activities on basic psychological parameters of humans acquire great importance. In this paper, we suggest the model that takes the parameters—motivation, attention, and anxiety—into account and improves educational achievement of students through Pilates exercises.

**Figure 1:**
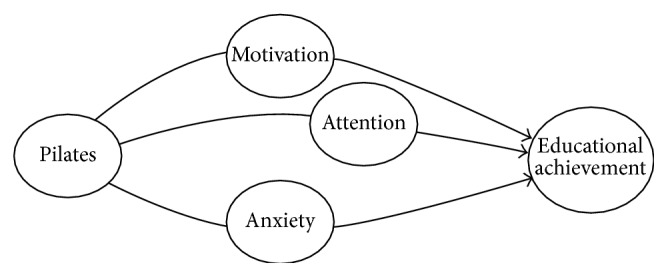


The structure that we have designed for modeling the effect of Pilates exercises on motivation, attention, anxiety, and educational achievement is presented in Figure 1.

There is broad agreement in the literature that the physical exercises and particularly Pilates exercises are associated with the motivation, attention, anxiety, and educational achievement [10–15]. The classical statistical approach is used today for finding the relationship between Pilates and above mentioned psychological parameters. Application of statistical techniques allows estimating the relationship between input (effect of Pilates) and output (achievement) from probabilistic point of view. This approach embraces only statistical uncertainty, but not fuzzy uncertainty inherent in psychological phenomena. Motivation of the application of *Z*-number theory to solve the presented problem is as follows.

(1) The concepts in the human brain for perceiving, recognizing, and categorizing natural phenomena are vague and imprecise. The boundaries of these parameters (such as low, moderate, or high levels of anxiety, motivation, and attention) are not exactly defined. Therefore, claiming that this emerges from them also becomes vague.
(2) *Z*-number theory allows us to estimate the relationship between input and output by using the concept of fuzzy information and partial reliability.
(3) *Z*-number based approach enables us to use uncertainty measures to quantify the ambiguity associated with prediction of psychological variables.

We will start with short introduction of basic terms: Pilates, motivation, attention, and anxiety.

## 2. Introduction

### 2.1. Benefits of Pilates

In developing his method, Pilates (founder of Pilates exercises) combines both Eastern and the Western concepts [10] by including mental focus and specific breathing of yoga with the Western physical exercise systems.

The mind-body approach is a basic of Pilates principles: centering, concentration, control, precision, flow, and breath [10, 11].

Pilates has the following physical, psychological, and social impacts [11–15].

1. Pilates exercise improves muscular strength, balance, posture, flexibility, and bone density and decreases back pain. The development of musculoskeletal fitness with long term resistance training is associated with enhanced cardiovascular function and musculoskeletal metabolism.
2. Pilates increases a cognitive function—Pilates is different than many other forms of exercise because it requires the mind to pay attention to what you are doing. Research shows that when required to think about how you are moving, your brain cells grow at a faster rate and your nervous system creates better connections throughout your body.
3. Pilates increases brain neurotransmitters, brain-derived neurotrophins that support neuronal differentiation and survival in the developing brain. Neurotrophins assure the survival of neurons in areas responsible for learning, memory, and higher thinking and, as an overall result, improve the overall quality of life.
4. Pilates increases blood flow and oxygen flow to the brain and raises levels of serotonin and endorphins—all of which help to reduce stress, anxiety, and fatigue and improve mood, motivation, and achievement.
5. Beyond academic achievement, many researchers connect Pilates to absenteeism, drop-out rate, and social communications of students.

### 2.2. Motivation

Motivation is defined as the process that initiates, guides, and maintains goal-oriented behaviors. It involves the biological, emotional, social, and cognitive forces that activate behavior. Motivation has been shown to positively influence study strategy and academic performance. Self-determination theory describes human motivation on a continuum categorized by amotivation, instincts, and extrinsic motivations [16].

Amotivation is the state of lacking the intention to act, whether resulting from not valuing the activity, not feeling capable of performing the task, or not expecting it to produce a desired outcome.

Extrinsic motivations are those that arise from outside of the individual and often involve rewards such as trophies, money, social recognition, or praise.

Intrinsic motivations are those that arise from within the individual, for the inherent satisfaction of the activity itself.

Pilates has effect on instinct motivation through the following mechanisms: improving total mood, body energy, self-esteem, psychological well-being, and vitality, reducing stress and anxiety, releasing certain neurotransmitters that alleviate physical and mental pain, and satisfying the basic psychological needs as competence, autonomy, and relatedness.

### 2.3. Anxiety

Anxiety is one of the major psychological variables which is considered as an important part of personality development. Anxiety is a psychological and physiological state characterized by somatic, emotional, cognitive, and behavioural components [17]. Anxiety generally helps in improving the performance of an individual. However, when anxiety becomes overwhelming, it may fall under the classification of anxiety disorder. This means anxiety should not cross its threshold value; otherwise it will reach up to an abnormal level.

American and European studies have found a negative correlation between anxiety and academic achievement.

Pilates has effect on anxiety through the following mechanisms: increasing body energy, sleep quality, attention, and concentration, releasing negatively thinking, improving blood and oxygen circulated to the brain, and relaxing muscles.

### 2.4. Attention

Attention was originally defined as processing one out of what seem several simultaneously possible objects or trains of thought. It implies withdrawal from some things in order to deal effectively with others [18].

Attention can be considered as a filter, in which many pieces of information come into the brain, but only one of these pieces of information is processed.

Pilates has effect on attention through the following mechanisms: concentration and precision are two main principles of Pilates; controlling a body movement by the brain; increasing sleep quality; relaxing of body; releasing negative thinking; decreasing the stress and anxiety.

## 3. Application of Fuzzy Logic and *Z*-Number Theory in Psychology Researches

The applications of fuzzy logic in psychology researches have been started since mid-1980 [19]. Averkin and Tarasov in [20] examined application of fuzzy modeling relation in psychology. Hesketh et al. considered [21] application of fuzzy graphical rating scale to the psychology. In [22] fuzzy logic based model of emotion is given. The role of fuzzy logic in psychological researches was examined in [23]. The researchers studied the relationship between motivation and anxiety using fuzzy logic and concluded that the fuzzy logic method has more advantages over statistical analysis to control uncertainties in data.

In this paper, we put a new approach for modeling of psychology processes through the *Z*-valuation concept. *Z*-information approach includes value of variable of interest and its reliability and has a major advantage for modeling such type of concepts. This approach enables us to use uncertainty measures to quantify the ambiguity associated with prediction of psychological parameters.

### 3.1. Preliminaries

A discrete *Z*-number [24] is an ordered pair $Z=(\tilde{A},\tilde{B})$, where $\tilde{A}$ is a discrete fuzzy number playing a role of a fuzzy constraint on values that a random variable *X* may take:

(1) $$\begin{array}{c} X\;\text{is}\;\tilde{A} \end{array}$$

and $\tilde{B}$ is a discrete fuzzy number with a membership function $\mu_{\tilde{B}}:\{b_{1},\ldots ,b_{n}\}\to [0,1],\;\;\{b_{1},\ldots ,b_{n}\}\subset [0,1]$, playing a role of a fuzzy constraint on the probability measure of $\tilde{A}$:

(2) $$\begin{array}{c} P\left(\tilde{A}\right)\;\text{is}\;\tilde{B}. \end{array}$$

A concept of a discrete *Z* ^+^-number is closely related to the concept of a discrete *Z*-number. Given a discrete *Z*-number $Z=(\tilde{A},\tilde{B}),\;\;Z^{+}$-number *Z* ^+^ is a pair consisting of a fuzzy number, $\tilde{A}$, and a random number *R*:

(3) $$\begin{array}{c} Z^{+}=\left(\tilde{A},R\right), \end{array}$$

where $\tilde{A}$ plays the same role as it does in a discrete *Z*-number $Z=(\tilde{A},\tilde{B})$ and *R* plays the role of the probability distribution *p*, such that $P(\tilde{A})=\sum_{i=1}^{n}\mu_{\tilde{A}}(x_{i})p(x_{i}),\;\;P(\tilde{A})\in \mathrm{supp}(\tilde{B})$.

## 4. Problem Solving

**Figure 2:**
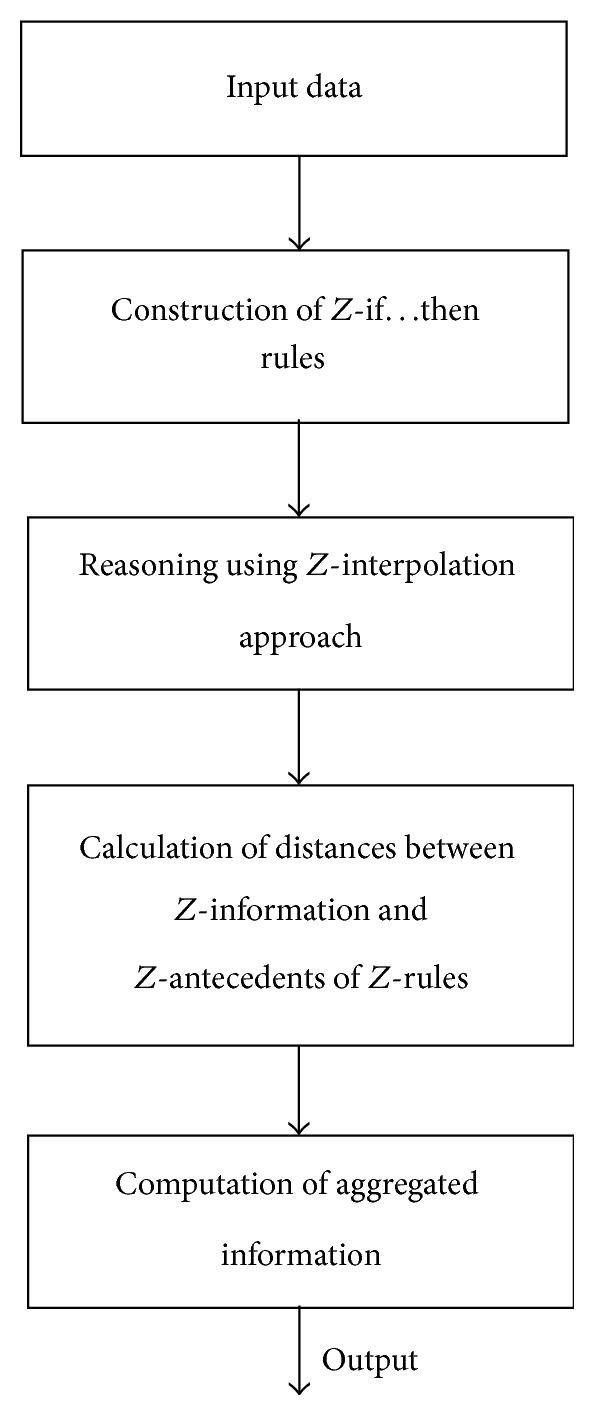
Flowchart diagram of *Z*-number modeling.

Flowchart diagram of *Z*-number based modeling is given in Figure 2.

Input raw data is created from the questionnaires (tests of motivation, attention, and anxiety) completed by students.

This data is imprecise and involves uncertainty related with process of completing (filling) of questionnaires. The results of measuring are processed as fuzzy variables with the different fuzzy subsets.

The relationships between educational achievement and the above mentioned psychological variables are presented as* Z-if…then* rules (Table 1) by the following linguistic variables: H—high; L—low; M—medium; G—good; E—excellence; U—usually; P—plausible; R—rare.

*Z*-rule base concept plays pivotal role in economics, decision making, forecasting, and other human centric systems functioning in *Z*-information environment. The *Z*-rule base is complete when for all the possible observations there exists at least one rule whose *Z*-antecedent part overlaps the current antecedent *Z*-valuation, at least partially. Otherwise, the *Z*-rule base is incomplete. In case that there is incomplete (sparse) *Z*-rule base, the classical reasoning methods based on compositional rule of inference (Zadeh [25], Mamdani [26], and R. A. Aliev and R. R. Aliev [27]) or Takagi and Sugeno [28] reasoning approach are not so effective to adapt generating an output for the observation covered by none of the rules. Consequently, we will use inference techniques which in the lack of matching rules can perform an approximate reasoning, namely, *Z*-interpolated methods.

A problem of *Z*-interpolation is an interpolation of of fuzzy is given below [24, 29].

Given the following *Z*-rules:

(4) $$\begin{array}{c} \text{If}\;X\;\text{is}\;\left(A_{X,1},B_{X,1}\right)\;\text{then}\;Y\;\text{is}\;\left(A_{Y,1},B_{Y,1}\right),\\ \text{If}\;X\;\text{is}\;\left(A_{X,2},B_{X,2}\right)\;\text{then}\;Y\;\text{is}\;\left(A_{Y,2},B_{Y,2}\right),\\ \vdots \\ \text{If}\;X\;\text{is}\;\left(A_{X,n},B_{X,n}\right)\;\text{then}\;Y\;\text{is}\;\left(A_{Y,n},B_{Y,n}\right) \end{array}$$

and the fact that

(5) $$\begin{array}{c} X\;\text{is}\;\left(A_{X},B_{X}\right), \end{array}$$

find the *Z*-value of *Y*.

The idea underlying the suggested interpolation approach is that the ratio of distances between the conclusion and the consequent parts is identical to ones between the observation and the antecedent parts. For *Z*-rules interpolation we have

(6) $$\begin{array}{c} Z_{Y}=\frac{\sum_{i=1}^{n}\left(1/\mathrm{dist}\left(Z_{X},Z_{X,i}\right)\right)Z_{Y,i}}{\sum_{k=1}^{n}\left(1/\mathrm{dist}\left(Z_{X},Z_{X,i}\right)\right)}, \end{array}$$

where dist is the distance between *Z*-numbers. As dist, method suggested in [29] can be used.

Let us consider the special case of the considered problem of *Z*-rules interpolation.

Given the *Z*-rules

(7) $$\begin{array}{c} \text{If}\;X\;\text{is}\;A_{X,1}\;\text{then}\;Y\;\text{is}\;\left(A_{Y,1},B\right),\\ \text{If}\;X\;\text{is}\;A_{X,2}\;\text{then}\;Y\;\text{is}\;\left(A_{Y,2},B\right),\\ \vdots \\ \text{If}\;X\;\text{is}\;A_{X,n}\;\text{then}\;Y\;\text{is}\;\left(A_{Y,n},B\right) \end{array}$$

and the fact that *X* is (*A*_*X*_, *B*_*X*_), find the value of *Y*.

For this case, formula (6) is reduced to

(8) ZY=∑i=1n1/dist⁡ZX,ZX,iZY,i∑k=1n1/dist⁡ZX,ZX,i=∑i=1n1/dist⁡AX,AX,iAY,i,B∑k=1n1/dist⁡AX,AX,i.

For example, using supremum metric *d* of fuzzy numbers will be

(9) ZY=∑i=1n1/dist⁡AX,AX,iAY,i,B∑k=1n1/dist⁡AX,AX,i=∑i=1n1/dAX,AX,iAY,i,B∑k=1n1/dAX,AX,i.

Taking into account that 1/*d*(*A*_*X*_, *A*_*X*,*i*_) is a scalar and applying the approach to multiplication of a *Z*-number by a scalar described in [30] we will have

(10) $$\begin{array}{c} Z_{Y}=\left(A_{Y},B\right),\;\text{with}\;A_{Y}=\frac{\sum_{i=1}^{n}\left(1/d\left(A_{X},A_{X,i}\right)\right)A_{Y,i}}{\sum_{k=1}^{n}\left(1/d\left(A_{X},A_{X,i}\right)\right)}. \end{array}$$

Let the knowledge base of 26 *Z*-rules of the following form be given:

(11) If  X1  is  Z^i1=Ai1,Bi1,If  X2  is  Z^i2=Ai2,Bi2,If  X3  is  Z^i3=Ai3,Bi3then  Y  is  Z^Y,i=AYi,BYi,kkkkkkikkkkki=1,…,26.

The considered *Z*-rules were described in terms of linguistic labels of *A*_*ij*_, *B*_*ij*_, given in Table 1.

Consider a problem of reasoning within the given *Z*-rules base by using *Z*-interpolation approach. Let the current input information be described by the following *Z*-numbers ${\hat{Z}}_{1}=(Z_{A_{1}},Z_{B_{1}}),\;\;{\hat{Z}}_{2}=(Z_{A_{2}},Z_{B_{2}}),\;\;{\hat{Z}}_{3}=(Z_{A_{3}},Z_{B_{3}})$:

(12) $$\begin{array}{c} Z_{A_{1}}=\frac{0.1}{0.3}+\frac{0.5}{0.35}+\frac{1}{0.4}+\frac{0.5}{0.45}+\frac{0.1}{0.5},\\ Z_{B_{1}}=\frac{0.1}{0.6}+\frac{0.5}{0.65}+\frac{1}{0.7}+\frac{0.5}{0.75}+\frac{0.1}{0.8},\\ Z_{A_{2}}=\frac{0.1}{0.2}+\frac{0.5}{0.25}+\frac{1}{0.3}+\frac{0.5}{0.35}+\frac{0.1}{0.4},\\ Z_{B_{2}}=\frac{0.1}{0.6}+\frac{0.5}{0.65}+\frac{1}{0.7}+\frac{0.5}{0.75}+\frac{0.1}{0.8},\\ Z_{A_{3}}=\frac{0.1}{0.62}+\frac{0.5}{0.67}+\frac{1}{0.7}+\frac{0.5}{0.73}+\frac{0.1}{0.8},\\ Z_{B_{3}}=\frac{0.1}{0.6}+\frac{0.5}{0.65}+\frac{1}{0.7}+\frac{0.5}{0.75}+\frac{0.1}{0.8}. \end{array}$$

*Z*-interpolation approach based reasoning consists of two main stages.

(1) For each rule compute distance *D*_*i*_ between the current input *Z*-information ${\hat{Z}}_{1}=(Z_{A_{1}},Z_{B_{1}}),\;\;{\hat{Z}}_{2}=(Z_{A_{2}},Z_{B_{2}}),\;\;{\hat{Z}}_{3}=(Z_{A_{3}},Z_{B_{3}})$ and *Z*-antecedents of *Z*-rules base ${\hat{Z}}_{i1}=(A_{i1},B_{i1}),\;\;{\hat{Z}}_{i2}=(A_{i2},B_{i2}),\;\;{\hat{Z}}_{i3}=(A_{i3},B_{i3})$ as follows:

(13) $$\begin{array}{c} D_{i}=\overset{3}{\underset{j=1}{\sum }}D\left({\hat{Z}}_{j},{\hat{Z}}_{ij}\right), \end{array}$$

where $D\left({\hat{Z}}_{j},{\hat{Z}}_{ij}\right)$ is the supremum metric:

(14) $$\begin{array}{c} D\left({\hat{Z}}_{j},{\hat{Z}}_{ij}\right)=d_{H}\left({\tilde{A}}_{j},{\tilde{A}}_{ij}\right)+d_{H}\left({\tilde{B}}_{j},{\tilde{B}}_{ij}\right), \end{array}$$

with

(15) $$\begin{array}{c} d_{H}\left({\tilde{A}}_{j},{\tilde{A}}_{ij}\right)=\sup\left\{d_{H}\left({A_{j}}^{\alpha },{A_{ij}}^{\alpha }\right)\;|\;0<\alpha \leq 1\right\},\\ d_{H}\left({\tilde{B}}_{j},{\tilde{B}}_{ij}\right)=\sup\left\{d_{H}\left({B_{j}}^{\alpha },{B_{ij}}^{\alpha }\right)\;|\;0<\alpha \leq 1\right\}. \end{array}$$

Consider computation of *D*_*i*_ for 1st and 15th rules. *Z*-antecedents of the 1st rule are *Z*-numbers ${\hat{Z}}_{11}=(Z_{A_{11}},Z_{B_{11}}),\;\;{\hat{Z}}_{12}=(Z_{A_{12}},Z_{B_{12}}),\;\;{\hat{Z}}_{13}=(Z_{A_{13}},Z_{B_{13}})$:

(16) $$\begin{array}{c} Z_{A_{11}}=\frac{1}{0.1}+\frac{0.75}{0.2}+\frac{0.5}{0.3}+\frac{0.25}{0.4}+\frac{0.1}{0.5},\\ Z_{B_{11}}=\frac{0.1}{0.7}+\frac{0.5}{0.75}+\frac{1}{0.8}+\frac{0.5}{0.85}+\frac{0.1}{0.9},\\ Z_{A_{12}}=\frac{1}{0.1}+\frac{0.75}{0.2}+\frac{0.5}{0.3}+\frac{0.25}{0.4}+\frac{0.1}{0.5},\\ Z_{B_{12}}=\frac{0.1}{0.7}+\frac{0.5}{0.75}+\frac{1}{0.8}+\frac{0.5}{0.85}+\frac{0.1}{0.9},\\ Z_{A_{13}}=\frac{0.1}{0.5}+\frac{0.25}{0.57}+\frac{0.5}{0.65}+\frac{0.75}{0.72}+\frac{1}{0.8},\\ Z_{B_{13}}=\frac{0.1}{0.7}+\frac{0.5}{0.75}+\frac{1}{0.8}+\frac{0.5}{0.85}+\frac{0.1}{0.9}. \end{array}$$

Thus, we need to compute $D_{1}=\sum_{j=1}^{3}D({\hat{Z}}_{j},{\hat{Z}}_{1j})$ according to (13), where $D({\hat{Z}}_{1},{\hat{Z}}_{11}),\;\;D({\hat{Z}}_{2},{\hat{Z}}_{12}),\;\;D({\hat{Z}}_{3},{\hat{Z}}_{13})$ are computed on the base of (14). We have obtained the results:

(17) DZ^1,Z^11=dHA~1,A~11+dHB~1,B~11DZ^1,Z^11=0.5+0.5=1,DZ^2,Z^12=1,DZ^3,Z^13=0.62.

Thus, the distance for 1st rule is

(18) $$\begin{array}{c} D_{1}=2.62. \end{array}$$

The inputs of the 15th rule ${\hat{Z}}_{15,1}=(Z_{A_{15,1}},Z_{B_{15,1}}),\;\;{\hat{Z}}_{15,2}=(Z_{A_{15,2}},Z_{B_{15,2}}),\;\;{\hat{Z}}_{15,3}=(Z_{A_{15,3}},Z_{B_{15,3}})$ are

(19) $$\begin{array}{c} Z_{A_{15,1}}=\frac{1}{0.1}+\frac{0.75}{0.2}+\frac{0.5}{0.3}+\frac{0.25}{0.4}+\frac{0.1}{0.5},\\ Z_{B_{15,1}}=\frac{0.1}{0.7}+\frac{0.5}{0.75}+\frac{1}{0.8}+\frac{0.5}{0.85}+\frac{0.1}{0.9},\\ Z_{A_{15,2}}=\frac{1}{0.1}+\frac{0.75}{0.2}+\frac{0.5}{0.3}+\frac{0.25}{0.4}+\frac{0.1}{0.5},\\ Z_{B_{15,2}}=\frac{0.1}{0.7}+\frac{0.5}{0.75}+\frac{1}{0.8}+\frac{0.5}{0.85}+\frac{0.1}{0.9},\\ Z_{A_{15,3}}=\frac{0.1}{0.5}+\frac{0.25}{0.57}+\frac{0.5}{0.65}+\frac{0.75}{0.72}+\frac{1}{0.8},\\ Z_{B_{15,3}}=\frac{0.1}{0.7}+\frac{0.5}{0.75}+\frac{1}{0.8}+\frac{0.5}{0.85}+\frac{0.1}{0.9}. \end{array}$$

Analogously, we computed the distance for 15th rule as

(20) DZ^1,Z^15,1=0.8,DZ^2,Z^15,2=1,DZ^3,Z^15,3=1.02,D15=2.82.

The distances computed for the rest of the rules are

(21) $$\begin{array}{c} D_{1}=2.62,\;\;D_{7}=3.02,\;\;D_{13}=2.9,\;\;D_{19}=3,\\ D_{25}=2.52,\;\;D_{2}=2.92,\;\;D_{8}=2.9,\;\;D_{14}=2.42,\\ D_{20}=2.92,\;\;D_{26}=2.52,\;\;D_{3}=3,\;\;D_{9}=2.72,\\ D_{15}=2.82,\;\;D_{21}=2.92,\;\;D_{4}=3.1,\;\;D_{10}=2.72,\\ D_{16}=2.82,\;\;D_{22}=2.82,\;\;D_{5}=3.1,\;\;D_{11}=2.32,\\ D_{17}=2.82,\;\;D_{23}=2.9,\;\;D_{6}=3.02,\;\;D_{12}=2.9,\\ D_{18}=3,\;\;D_{24}=2.9. \end{array}$$

(2) Computation of the aggregated output *Z*_*Y*_ for *Z*-rules base by using linear *Z*-interpolation are as follows:

(22) $$\begin{array}{c} Z_{Y}=\overset{n}{\underset{i=1}{\sum }}w_{i}Z_{Y,i},\;w_{i}=\frac{1}{D_{i}\sum_{k=1}^{n}\left(1/D_{k}\right)}. \end{array}$$

Thus, we need to compute convex combination of outputs *Z*_*Y*_*i*__ of the rules base. The outputs of the *Z*-rules base are as follows:

(23) $$\begin{array}{c} Z_{A_{Y_{1}}}=\frac{1}{0.1}+\frac{0.75}{0.2}+\frac{0.5}{0.3}+\frac{0.25}{0.4}+\frac{0.1}{0.5},\\ Z_{B_{Y_{1}}}=\frac{0.1}{0.6}+\frac{0.5}{0.65}+\frac{1}{0.7}+\frac{0.5}{0.75}+\frac{0.1}{0.8},\\ \vdots \\ Z_{A_{Y_{15}}}=\frac{0.1}{0.1}+\frac{0.5}{0.3}+\frac{1}{0.5}+\frac{0.5}{0.62}+\frac{0.1}{0.75},\\ Z_{B_{Y_{15}}}=\frac{0.1}{0.1}+\frac{0.5}{0.15}+\frac{1}{0.2}+\frac{0.5}{0.25}+\frac{0.1}{0.3},\\ \vdots \end{array}$$

Therefore, the aggregated output *Z*_*Y*_ is defined as

(24) $$\begin{array}{c} Z_{Y}=0.042Z_{Y_{1}}+0.037Z_{Y_{2}}+\cdots +0.038Z_{Y_{26}}=\left(A_{Y},B_{Y}\right). \end{array}$$

We have obtained the following result:

(25) AY=0.10.395+0.50.518+10.627+0.50.644+0.250.656,BY=0.10.7+0.50.75+10.8+0.50.85+0.10.9.

In accordance with codebook we have the following. Achievement is “medium” with the reliability “usually.”

The analysis shows that there are no significant differences between the mean of results obtained by conventional statistical method and *Z*-number based modeling. The standard deviations of the outputs estimated by statistical method and *Z*-number based modeling show significant differences of results. The less variance in the *Z*-number based modeling method is related with the ability of this method to control uncertainty in data.

## 5. Conclusion

In this paper we have suggested a new approach for modeling of the effect of the Pilates exercises on students' motivation, attention, anxiety, and educational achievement. The uncertainty of data related with cognitive measuring of psychological parameters and their partial reliability have been promoted for the first time application of “*Z-if…then* rules” for modeling the considered relationship.

We used an inference techniques for approximate reasoning based on *Z*-interpolation method suggested by Zadeh to embrace incomplete and lack of matching rules.

Computer simulation proves adequacy of the model.

## Figures and Tables

**Table 1:** *Z-if…then* rules.

Number of rules	If			Then
	Motivation	Attention	Anxiety	Achievement
1	(L, U)	(L, U)	(H, U)	(L, U)
2	(L, U)	(M, U)	(M, U)	(M, U)
3	(L, U)	(M, U)	(L, U)	(G, U)
4	(L, U)	(H, U)	(L, U)	(G, U)
5	(L, U)	(H, U)	(L, U)	(E, U)
6	(L, U)	(L, U)	(M, U)	(L, U)
7	(L, U)	(H, U)	(M, U)	(G, U)
8	(M, U)	(H, U)	(L, U)	(E, P)
9	(M, U)	(M, U)	(M, U)	(G, U)
10	(M, U)	(M, U)	(M, U)	(M, U)
11	(M, U)	(M, U)	(H, U)	(L, U)
12	(M, U)	(H, U)	(L, U)	(G, U)
13	(M, U)	(H, U)	(L, U)	(E, U)
14	(M, U)	(H, U)	(H, U)	(M, U)
15	(M, U)	(H, U)	(M, U)	(M, U)
16	(M, U)	(H, U)	(M, U)	(G, U)
17	(M, U)	(H, U)	(M, U)	(E, U)
18	(H, U)	(H, U)	(L, U)	(E, R)
19	(H, U)	(H, U)	(L, U)	(G, U)
20	(H, U)	(H, U)	(M, U)	(E, U)
21	(H, U)	(H, U)	(M, U)	(G, U)
22	(H, U)	(M, U)	(M, U)	(G, U)
23	(H, U)	(M, U)	(L, U)	(G, U)
24	(H, U)	(M, U)	(L, U)	(M, U)
25	(H, U)	(L, U)	(H, U)	(L, U)
26	(H, U)	(L, U)	(M, U)	(M, U)
27	(H, U)	(L, U)	(L, U)	(L, U)
28	(H, U)	(L, U)	(L, U)	(M, U)
